# Use and Understanding of Anonymization and De-Identification in the Biomedical Literature: Scoping Review

**DOI:** 10.2196/13484

**Published:** 2019-05-31

**Authors:** Raphaël Chevrier, Vasiliki Foufi, Christophe Gaudet-Blavignac, Arnaud Robert, Christian Lovis

**Affiliations:** 1 Division of Medical Information Sciences University Hospitals of Geneva Geneva Switzerland; 2 Faculty of Medicine University of Geneva Geneva Switzerland

**Keywords:** anonymization, anonymisation, de-identification, deidentification, pseudonymization, privacy, confidentiality, secondary use, data protection, scoping review

## Abstract

**Background:**

The secondary use of health data is central to biomedical research in the era of data science and precision medicine. National and international initiatives, such as the Global Open Findable, Accessible, Interoperable, and Reusable (GO FAIR) initiative, are supporting this approach in different ways (eg, making the sharing of research data mandatory or improving the legal and ethical frameworks). Preserving patients’ privacy is crucial in this context. De-identification and anonymization are the two most common terms used to refer to the technical approaches that protect privacy and facilitate the secondary use of health data. However, it is difficult to find a consensus on the definitions of the concepts or on the reliability of the techniques used to apply them. A comprehensive review is needed to better understand the domain, its capabilities, its challenges, and the ratio of risk between the data subjects’ privacy on one side, and the benefit of scientific advances on the other.

**Objective:**

This work aims at better understanding how the research community comprehends and defines the concepts of de-identification and anonymization. A rich overview should also provide insights into the use and reliability of the methods. Six aspects will be studied: (1) terminology and definitions, (2) backgrounds and places of work of the researchers, (3) reasons for anonymizing or de-identifying health data, (4) limitations of the techniques, (5) legal and ethical aspects, and (6) recommendations of the researchers.

**Methods:**

Based on a scoping review protocol designed a priori, MEDLINE was searched for publications discussing de-identification or anonymization and published between 2007 and 2017. The search was restricted to MEDLINE to focus on the life sciences community. The screening process was performed by two reviewers independently.

**Results:**

After searching 7972 records that matched at least one search term, 135 publications were screened and 60 full-text articles were included. (1) Terminology: Definitions of the terms de-identification and anonymization were provided in less than half of the articles (29/60, 48%). When both terms were used (41/60, 68%), their meanings divided the authors into two equal groups (19/60, 32%, each) with opposed views. The remaining articles (3/60, 5%) were equivocal. (2) Backgrounds and locations: Research groups were based predominantly in North America (31/60, 52%) and in the European Union (22/60, 37%). The authors came from 19 different domains; computer science (91/248, 36.7%), biomedical informatics (47/248, 19.0%), and medicine (38/248, 15.3%) were the most prevalent ones. (3) Purpose: The main reason declared for applying these techniques is to facilitate biomedical research. (4) Limitations: Progress is made on specific techniques but, overall, limitations remain numerous. (5) Legal and ethical aspects: Differences exist between nations in the definitions, approaches, and legal practices. (6) Recommendations: The combination of organizational, legal, ethical, and technical approaches is necessary to protect health data.

**Conclusions:**

Interest is growing for privacy-enhancing techniques in the life sciences community. This interest crosses scientific boundaries, involving primarily computer science, biomedical informatics, and medicine. The variability observed in the use of the terms de-identification and anonymization emphasizes the need for clearer definitions as well as for better education and dissemination of information on the subject. The same observation applies to the methods. Several legislations, such as the American Health Insurance Portability and Accountability Act (HIPAA) and the European General Data Protection Regulation (GDPR), regulate the domain. Using the definitions they provide could help address the variable use of these two concepts in the research community.

## Introduction

### Background

In 2003, the National Institutes of Health (NIH) released its final statement on sharing research data. The NIH made the provision of a data-sharing plan mandatory for any funding starting at US $500,000 per year [1]. This statement, among other published work [2-5], accelerated the sharing of research data worldwide in parallel to the growing availability of data and information technologies. In this context, the research community gained an unprecedented capacity to access and analyze large amounts of health data, originating partly from nonresearch sources. The use of medical data for a different purpose than the one it was initially collected for is commonly called “secondary use of medical data” [3]. This particular use of health data is subject to technical and semantic problems as well as legal, ethical, and societal concerns. To comply with the legal and ethical principles, researchers have two main options to access and use medical data for a secondary purpose [6]. One option is to gain patients’ consent specifically for the new purpose of their research. This is generally complicated and costly [7]. Alternatively, they can de-identify the data, since the law permits the disclosure of clinical information if it has been correctly de-identified [8]. Institutional review boards (IRBs) generally waive the need for consent in this situation [9]. The existence of the second option gives de-identification and anonymization a pivotal role in biomedical research. Consequently, the availability of reliable techniques to protect privacy becomes essential for the research community to leverage the secondary use of medical data [10].

Despite all efforts, an important gap still exists between the needs and the access to massive data in science. Large collaborative data-sharing projects are somehow below expectations and the research community is calling for improved open data and open science [11]. Some authors have proposed explanations as to why data sharing is more complicated in practice than in theory [3]. An article has considered the influence of policies and of our capacity to protect the data on our ability to share it [12]. Reviews have been published on the techniques and systems aiming at protecting health data privacy [13,14]; one has collected and studied the known re-identification attacks on health data [15], and another has looked specifically into the security and privacy issues related to electronic health records [16]. Various techniques aim at protecting the medical data subjects’ privacy. Those that do not strictly represent an anonymization or de-identification process are not part of the scope of this review. Cryptography, privacy-preserving record linkage [17], and differential privacy [18] are among these techniques.

Although advanced probabilistic privacy-enhancing methods have been studied and applied for over three decades in other areas [19], their application to medical data is a fairly recent interest for the biomedical research community. A striking example is the late introduction of *data anonymization* (2016) and other central concepts of health data privacy (eg, *personally identifiable information*) in the Medical Subject Headings (MeSH) thesaurus of the US National Library of Medicine. Over the last few years, a great amount of expert literature was produced on anonymization and de-identification techniques for medical data. However, publications providing the readers with a broad understanding of these techniques, and addressing their application in life sciences and clinical research in a comprehensive way, are lacking. As a consequence, the fundamental concepts remain either unknown to the research community or difficult to comprehend. Adding to the confusion, a well-documented and long-standing ambiguity exists in the vocabulary used by those who contribute to the practice [20,21]. In particular, the terms *de-identification* and *anonymization* have been used with different meanings by researchers. De-identification is frequently, but not exclusively, used in the biomedical literature to refer to rule-based techniques. These techniques often apply the rules provided in the *Safe Harbor* method of the American legislation (ie, the Health Insurance Portability and Accountability Act [HIPAA]). On the other hand, anonymization is commonly, but not exclusively, used in the biomedical literature to refer to statistical or probabilistic techniques. Turning to the legislations to clarify the meaning of these terms can bring further confusion. Although researchers tend to use two different terms—de-identification and anonymization—to refer to one approach or the other, the American law itself regards both approaches (ie, rule-based or probabilistic) as ways to achieve de-identification. The first follows the *Safe Harbor* method—§164.514(b)(2)—and the second follows the *Expert Determination* method—§164.514(b)(1). The European legislation (ie, the General Data Protection Regulation [GDPR]), on the other hand, does not use either of the terms.

A growing number of health care data breaches are being reported [22], some resulting directly from a failure to anonymize or de-identify the data properly [23]. In this context, it seems essential to review the literature published on this rapidly evolving domain to inform researchers, doctors, lawmakers, and the public about instruments that are becoming indispensable to researchers. This is true, especially since these instruments have bearing on subjects of paramount importance for the future of medical research, namely, data sharing, data privacy, and public trust in health care research and institutions.

### Objectives

The aim of this work is to better understand how the life sciences research community defines, comprehends, and uses the concepts of de-identification and anonymization. Providing a broad perspective on the field, this review should also contextualize these concepts and their application in today’s biomedical research domain. To attain these goals, the reviewers identified six key aspects to study, which are presented in Textbox 1. These aspects are central to this work; they guided the data collection and they structured the Results section.

**Objectives: subjects of focus for this scoping review.**

1. Vocabulary, definitions, and understandings of the terms *anonymization* and *de-identification*.
2. Authors’ backgrounds and places of work.
3. Reasons for anonymizing or de-identifying health data.
4. Limitations of anonymization and de-identification techniques.
5. Legal and ethical implications of the practice.
6. Experts’ recommendations.

## Methods

### Overview

Scoping reviews represent an increasingly popular type of review [24], which allows for the mapping of concepts in a field of interest. They are intended to study complex and overlapping domains, particularly when they have not been reviewed comprehensively before [25]. To conduct this work, the authors used the guidance proposed by the Joanna Briggs Institute for the conduct of scoping reviews [26].

The first step of the scoping review process was to perform a preliminary, nonsystematic survey of the literature regarding de-identification and anonymization. This survey identified the key concepts, the concerns, the challenges, and the gaps in the domain. This information was used to define the study’s objectives and to design the study protocol.

### Article Identification and Selection

#### Search Strategy

Aiming to focus this work on the life sciences researchers’ community, the articles were sourced selectively from one database: MEDLINE [27]. To maximize the sensitivity and specificity of the search query, several strategies were tested and implemented. The terms “de-identification” and “anonymization”; their lexical variants (eg, “de-identif*,” and “anonymi*”); and their spelling variants (eg, “deidentified” without hyphen and “anonymisation” in British English) were used. Alternative spellings were proven effective in a previous literature review on the same topic [28]. Numerous candidate terms were tested, here are some examples: “privacy protection,” “data protection,” “confidentiality,” “personal data,” “medical data,” ”re-identification,” and “breaches”. None of these terms increased the sensitivity of the search compared to the terms “de-identification,” “anonymization,” and their variants. The same conclusion was reached regarding the use of the MeSH-controlled vocabulary. Finally, search-field descriptors—[ti] (Title) and [tiab] (Title/Abstract)—were used, and the terms were combined between themselves using Boolean operators. A full description of the search query is provided in the Results section.

#### Inclusion Criteria

This work analyses the literature published between November 1, 2007, and November 1, 2017. Only original research articles and review articles available in full text through the University of Geneva’s library network were considered. Additionally, publications had to meet at least one of the following three criteria to be included:

1. The subject of the article is the process of rendering medical data as less identifiable using computer techniques (ie, de-identification or anonymization).
2. The article focuses on sharing medical data; however, protecting the patients’ privacy using computer techniques is also discussed.
3. The article presents legal and ethical aspects of sharing medical data, and the concept of de-identification or anonymization is discussed.

#### Exclusion Criteria

The literature addressing certain data types was excluded: video recordings, photographic images, radiological images, and geolocation data. This decision was made on the basis of the information found during the preliminary literature survey [28,29] and was confirmed after discussions with experts. Short reports, posters, and editorials were also excluded.

### Data Collection

Based on the list of six objectives (see Textbox 1), information categories were defined (see Table 1). Quantitative and qualitative data were collected from the articles. Quantitative information was extracted for certain categories and statistical analysis was performed on this data. Qualitative information was collected for the categories not suited to quantitative analysis. This second approach was nonetheless important, as it allowed us to bring together the views of some experts and to identify consensus or disagreements.

**Table 1:** Categories of information used to collect quantitative and qualitative data from the reviewed articles.

Type of data	Categories of information
Quantitative	Journal Year of publication Author(s) Authors’ backgrounds Authors’ places of work Presence of the terms “de-identification” and “anonymization” Definitions of the terms “de-identification” and “anonymization” Meanings given to the terms “de-identification” and “anonymization”
Qualitative	Purposes of de-identification and anonymization Limitations of the privacy-enhancing techniques Ethical or legal considerations Suggestions and recommendations Data utility and information loss Data sharing in biomedical research Types of data subjected to anonymization or de-identification Public opinion on privacy-enhancing techniques and health data sharing

To determine the backgrounds of the authors, points were attributed to domains (medicine, computer science, law, etc) according to each author’s professional affiliation and academic qualifications. Up to three authors were included per publication (ie, first, second, and last author), based on a previous research study, which showed that the most significant contributions were made by these authors [30]. All publications included in the review were considered. The information about the authors was collected manually from the articles, from the authors’ or organizations’ websites, and from other sources, such as Google Scholar, Open Researcher and Contributor ID (ORCID), ResearchGate, etc.

## Results

### Study Selection and Characteristics

**Figure 1:**
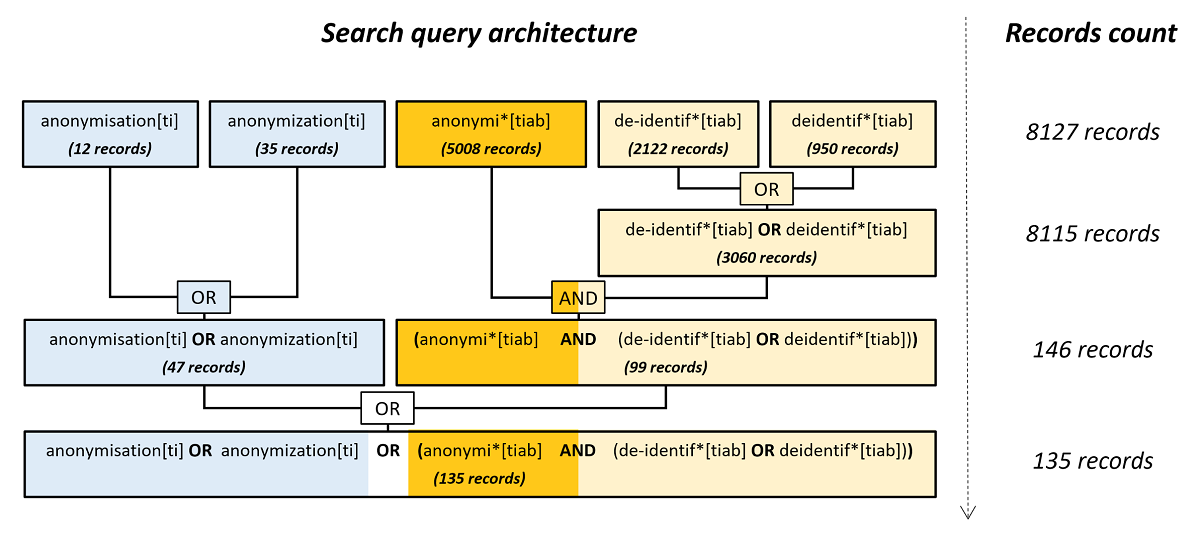
Architecture and breakdown of the search query with the number of records at each level. [ti]: Title; [tiab]: Title/Abstract.

The literature search retrieved 135 articles from the sizeable number of existing records—7972 after the removal of duplicates—containing at least one of the search terms used. The breakdown of the search query shows the number of records at each level (see Figure 1).

**Figure 2:**
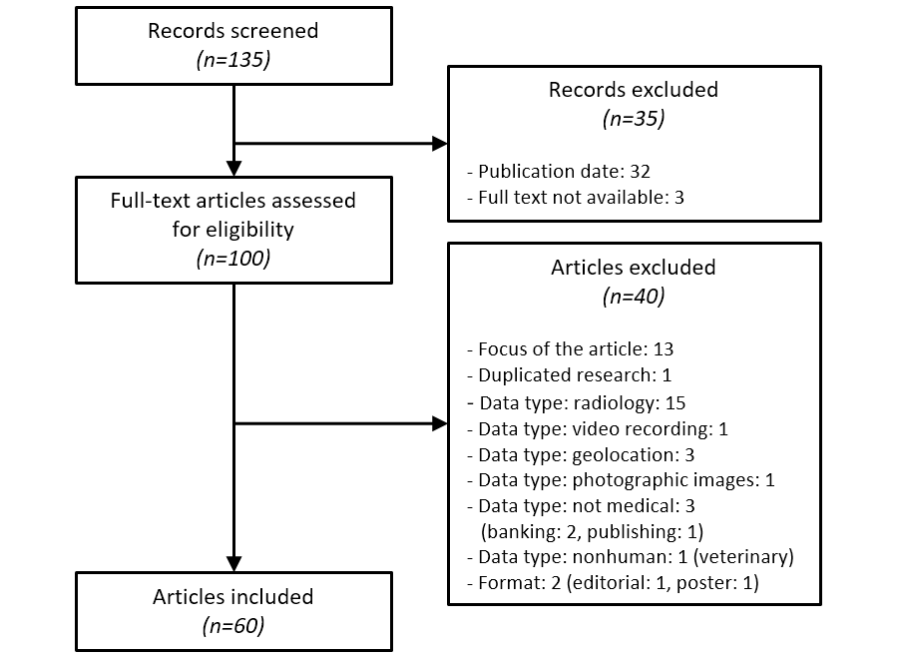
Preferred Reporting Items for Systematic Reviews and Meta-Analyses (PRISMA) flow diagram for the scoping review process (ie, screening, eligibility, and inclusion).

The search query identified 135 records in MEDLINE corresponding to the keyword search; the records were then manually screened according to the Preferred Reporting Items for Systematic Reviews and Meta-Analyses (PRISMA) methodology (see Figure 2). Among them, 103 records were in the considered time frame. Three records were excluded because the full text could not be retrieved. An additional 40 articles were excluded based on the focus of the paper, the data type considered, or the publication type. During this process, five records raised questions about their potential eligibility. A third reviewer was involved to reach consensus.

The process resulted in the inclusion of 60 articles; the list is available in Multimedia Appendix 1. The 60 articles came from 32 different scientific journals. Corrected for the five journals that did not have a registered impact factor in 2017, the average impact factor of the journals included in this review was 2.859, ranging from 9.504 for the highest to 0.304 for the lowest, with a median of 2.766. More than a third of the articles (23/60, 38%) were published after 2015 (see Table 2).

**Table 2:** Characteristics of the 60 articles included in the review and of the journals where they were published.

Characteristics		Count (N=60), n (%)
**Year of publication**		
	2008-2009	5 (8)
	2010-2011	11 (18)
	2012-2013	10 (17)
	2014-2015	11 (18)
	2016-2017	23 (38)
**Scientific domains of the journals**		
	Biomedical informatics	32 (53)
	Engineering	8 (13)
	Public health, methodology, and epidemiology	6 (10)
	Bioethics and law & health policies	5 (8)
	Medicine: biomedical sciences	5 (8)
	Medicine: clinical	4 (7)

### Vocabulary and Definitions

Half of the articles (29/60, 48%) provided a definition of de-identification or anonymization (see Table 3).

The attempts at defining the terms were rare, and the definitions often vague, inconsistent, or even contradictory (see Table 4). Referring to the HIPAA *Safe Harbor* method of de-identification, one article correctly recommended the removal of 18 types of protected health information (PHI) [13]. Another suggested the removal of 17 types of PHI [31]. Regarding the processing of the types of PHI, one article proposed to “hide” or “remove” them [13], while another suggested to “extract” or “replace” them with pseudonyms [32]. Concerning anonymization, the variability was similar. One article presented the process as the removal of the patients’ names [33]. Another considered it a much more radical alteration of the data, which would be virtually impossible to reverse [28].

Conflicting representations of de-identification and anonymization were uncovered (see Textbox 2). In some articles, the terms are used interchangeably to refer to the same concept [34-37], while in others they outline strictly different processes [13,19,28,38].

The researchers’ representations of de-identification and anonymization, as similar or different concepts, were counted from the reviewed articles to determine whether or not there was a consensus among the experts. The results are presented in Table 5. The 38 authors who used both terms were evenly split between those who considered the two notions to be identical (19/60, 32%) and those who considered them to be different (19/60, 32%).

The 19 researchers who only used or discussed one concept in the core of their articles mentioned the second one in the keywords or title. From the reviewers’ perspective, this finding reinforces the idea that de-identification and anonymization are synonyms in many people’s minds.

**Table 3:** Presence of definitions for the terms *de-identification* or *anonymization* in the reviewed articles.

	Terms with definitions	Count (N=60), n (%)
	De-identification	26 (43)
	Anonymization	12 (20)
	Both	9 (15)
	None	31 (52)

**Table 4:** Examples of attempts to define the terms *de-identification* or *anonymization*.

Terms	Definitions
De-identification	“For clinical data to be considered de-identified, the HIPAA ‘Safe Harbor’ technique requires 18 data elements (called PHI: Protected Health Information) to be removed...de-identification only means that explicit identifiers are hidden or removed.” [13] “Under Safe Harbor, data are considered de-identified if 17 listed types of identifiers are removed.” [31] “de-identification where explicit identifiers (e.g., Protected Health Information [PHI] elements) are extracted or replaced with ‘pseudonyms’” [32] “De-identification of medical record data refers to the removal or replacement of personal identifiers so that it would be difficult to reestablish a link between the individual and his or her data. Although a de-identified dataset may contain an encrypted patient identifier with which authorized individuals could relink a patient with his or her dataset, this dataset must not contain data that will allow an unauthorized individual to infer a patient’s identity from the existing data elements.” [28]
Anonymization	“The anonymization consists in removing the patients’ names from the records: unfortunately, other pieces of information enable to identify the patients.” [33] “anonymization implies that the data cannot be linked to identify the patient” [13] “the process of rendering data into a form which does not identify individuals and where identification is not likely to take place” [10] “Data anonymization is the process of conditioning a dataset such that no sensitive information can be learned about any specific individual.” [19] “Anonymization refers to the irreversible removal of the link between the individual and his or her medical record data to the degree that it would be virtually impossible to reestablish the link.” [28]

**Discrepancies in understanding and using de-identification and anonymization in relation to each other.**

Anonymization = de-identification:

- “Access to de-identified (anonymized) health records would in many cases be sufficient.” [34]
- “Anonymization: Redaction, perturbation, or generalization of those attributes that could be used, alone or in combination, to associate a given record with a specific person. Also called ‘de-identification.’” [35]
- “Recent renewed interest in de-identification (also known as ‘anonymisation’) has led to the development of a series of systems in the United States with very good performance on challenge test sets.” [36]
- “As has been seen, the European regime for privacy does not require the de-identification (anonymization) of personal data used in genomic databases or biobanks.” [37]

Anonymization ≠ de-identification:

- “we note that a recent analysis of matching attacks against a large, public, de-identified (although not anonymized) dataset independently came up” [19]
- “Anonymization and de-identification are often used interchangeably, but de-identification only means that explicit identifiers are hidden or removed, while anonymization implies that the data cannot be linked to identify the patient (i.e. de-identified is often far from anonymous).” [13]
- “De-identification of medical record data refers to the removal or replacement of personal identifiers so that it would be difficult to reestablish a link between the individual and his or her data...Anonymization refers to the irreversible removal of the link between the individual and his or her medical record data to the degree that it would be virtually impossible to reestablish the link.” [28]
- “The term ‘anonymization’ is not identical to ‘de-identification.’ De-identification is the removal of attributes known to increase the risk of identification, and this can be seen as a preliminary step for producing anonymous data. It requires, however, a further assessment as to whether the de-identification process achieves anonymization.” [38]

**Table 5:** Researchers’ understanding of de-identification and anonymization as similar or different concepts.

Use of the terms in the articles	Count (N=60), n (%)
Only use or discuss one concept	19 (32)
De-identification and anonymization are two different concepts	19 (32)
De-identification and anonymization are used interchangeably	19 (32)
Ambiguous with regard to the meaning of both terms	3 (5)

### Authors’ Backgrounds and Places of Work

Applying the scoring system presented in the Methods section, we counted 163 authors for the 60 publications. A total of 248 background points were attributed to 19 different domains (see Table 6).

The first seven fields represent 90% of the researchers’ backgrounds. On average, one researcher was awarded 1.52 research field points. A total of 14 researchers published more than one article (ie, 2-8 articles). Out of 14 prolific authors, 13 (93%) had a background in the three leading domains. Removing the duplicates revealed 121 unique authors. The number of domains and their ranking remained unchanged with and without duplicates, with a slightly smaller gap between the first three domains and the others when duplicates were removed. The background of 7 authors could not be found; this represents a margin of error of 4.3%.

**Figure 3:**
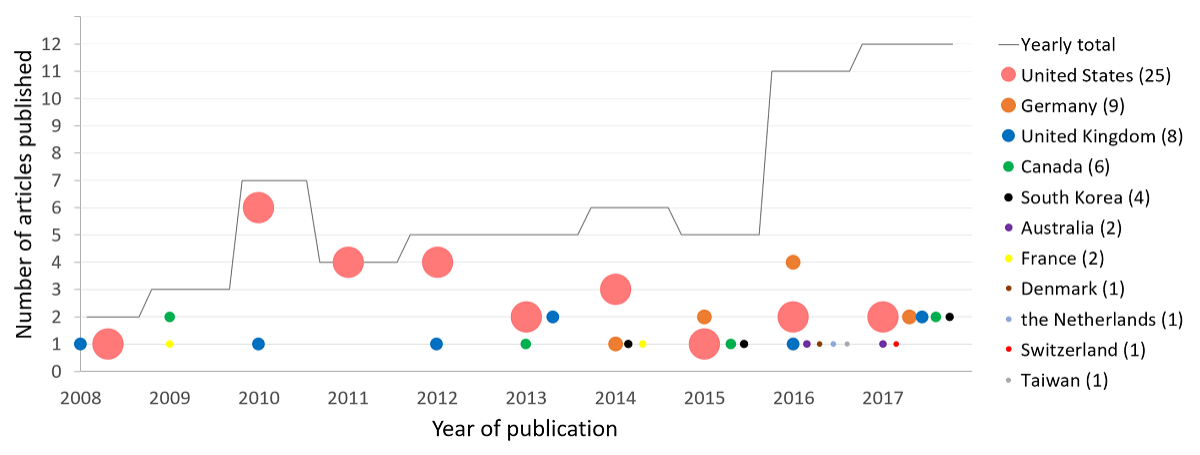
Representation of the 60 publications according to the date of publication, the number of articles per year, and the authors’ locations. The size of the discs used on the graph represents each country’s contribution in number of articles over the studied period (10 years). The exact count is shown between brackets next to each country’s name.

Regarding the place of work, the United States was the largest contributor with 25 articles (25/60, 42%), followed by Germany, the United Kingdom, and Canada combined (23/60, 38%). The predominance of publications from the US-based research groups is noticeable particularly between 2010 and 2012. After this period, their contribution decreases in absolute number and, more importantly, in relation to other groups, due to the arrival of new groups from 2014 and the rapid growth of publications on the topic of anonymization and de-identification. As a result, the leading position that American researchers (ie, Canada and the United States) held until 2013 was caught up to by researchers from other countries in 2014. Since 2015, European groups have been publishing an equal or greater number of articles than the Americans on this topic (see Figure 3).

### Purpose of Anonymization and De-Identification

Most often, the authors mentioned the secondary use of medical data without specification as to the purpose of their research. When specified, their objective was to enable and support biomedical research [7,32,39-41]. Regarding the research domains, *genetics and genomics* [42-49] were the most frequently cited, followed by *personalized health and precision medicine* [48,50-52]. Improvement in the domains of *epidemiology* and *public health surveillance and reporting* were among other anticipated benefits of developing privacy protection techniques [8,53]. The protection of privacy was implicit in most projects but was also explicitly cited as a standalone objective in some publications [50,54]. Complying with regulations and policies was a motivation expressed by certain authors [46,55]. Several other reasons were found, as shown in Textbox 3.

**Table 6:** Background points awarded to the authors of the reviewed articles. The authors are separated by authorship position: first, second, and last.

Research field	First author (N=92), n (%)	Second author (N=72), n (%)	Last author (N=84), n (%)	Total count (N=248), n (%)
Computer science	36 (14)	26 (10)	29 (12)	91 (36.7)
Biomedical informatics	16 (6)	15 (6)	16 (6)	47 (19.0)
Medicine (MD^a^)	13 (5)	9 (4)	16 (6)	38 (15.3)
Epidemiology and statistics	6 (2)	3 (1)	7 (3)	16 (6.5)
Mathematics and biomathematics	6 (2)	5 (2)	5 (2)	16 (6.5)
Law	3 (1)	3 (1)	2 (1)	8 (3.2)
Psychology	2 (1)	3 (1)	2 (1)	7 (2.8)
Linguistics	2 (1)	0 (0)	2 (1)	4 (1.6)
Project management	1 (0)	1 (0)	1 (0)	3 (1.2)
Bioethics and humanities	1 (0)	2 (1)	0 (0)	3 (1.2)
Public health	1 (0)	0 (0)	1 (0)	2 (0.8)
Neuroscience	2 (1)	0 (0)	0 (0)	2 (0.8)
Behavioral economy	0 (0)	2 (1)	0 (0)	2 (0.8)
Journalism	1 (0)	1 (0)	0 (0)	2 (0.8)
Biology and microbiology	1 (0)	0 (0)	1 (0)	2 (0.8)
Physics	1 (0)	1 (0)	0 (0)	2 (0.8)
Health care administration	0 (0)	0 (0)	1 (0)	1 (0.4)
Ecology and evolution	0 (0)	1 (0)	0 (0)	1 (0.4)
Business (MBA^b^)	0 (0)	0 (0)	1 (0)	1 (0.4)

^a^MD: Doctor of Medicine.

^b^MBA: Master of Business Administration.

**Additional reasons expressed by experts for de-identifying or anonymizing health data.**

1. Publication in biomedical journals [56].
2. Teaching [34].
3. Spontaneous reporting systems to collect adverse drug events [57].
4. Limiting the administrative burden of consent in research [7,38].
5. Facilitating clinical trial data publication [35].
6. Facilitating population screening programs [58].
7. Enabling the creation of medical text corpora for natural language processing (NLP) research and development [37].
8. Protecting particularly sensitive information (eg, mental health data) [59].
9. Producing reports on prescribing patterns and drug utilization and to perform economic studies [60].
10. Performing comparative effectiveness studies [45].

### Limitations of Anonymization and De-Identification Techniques

#### Technical and Operational Limitations

Anonymization and de-identification are time-consuming tasks, particularly when textual data is concerned [61]. The necessity for manual intervention is seen as a weakness that leaves room for human error and contributes to lengthening the procedure [62]. The difficulty in generalizing and scaling the de-identification and anonymization procedures, as well as the absence of broadly accepted metrics to judge their results, are recurrent concerns raised in publications [8,13,38,39,60]. The complexity of these procedures depends on the type of information involved. Structured information (eg, tabular data) is generally easier to process than unstructured information (eg, textual data) [29]. Specific types of information (eg, diagnoses of rare diseases [60]) are more identifying than others. Some types are even considered identifying by nature (eg, large genome sequencing) and presumably impossible to render anonymous [38,63]. More generally, balancing the probability of re-identification with the amount of distortion applied to the data is seen as a challenge [7,59]. Unable to overcome the interdependence between data quality and data identifiability, one has to be compromised for the other: “no existing anonymization algorithm provides both perfect privacy protection and perfect analytic utility” [19]. The re-identification risk depends on the availability of additional information. Using data linkage techniques, the presence of individuals in the protected dataset can be revealed and their personal information re-identified [51,64,65]. Because the amount of information available for comparison can only be estimated, the re-identification risk will always remain an estimate [66]. Additionally, this risk will increase over time [13,48]. These inherent weaknesses have led some researchers to express doubts about the reliability of anonymization or de-identification techniques [35,66,67].

#### Limitations in Accessibility and Governance

The substantial cost and the limited access to trained professionals are seen as hindrances for institutions wanting to share their data [29,33,66]. Disparities in the availability of anonymization and de-identification systems between English-speaking countries and the rest of the world is expressed by certain authors [33,40]. Textual data is primarily concerned by this problem with a critical need for natural language processing (NLP) systems in varied languages [36,68]. Authors report the lack of practical guidelines and training to assist the researchers [31,69]. They also report an absence of a consensus “regarding the effective governance of secondary research uses, beyond adherence to the terms of informed consent” [70]. Finally, several researchers point out the confusion affecting the terminology as a flaw in itself, increasing the risk of re-identification through misconceptions and misunderstandings [38,71].

### Legal and Ethical Implications

#### General

Privacy laws and regulations provide the legal framework for the collection, processing, and sharing of personal data [71]. Differences exist between nations in the definitions, approaches, and legal practices [66]. Commonly, legal experts agree that relying on legislation alone to protect privacy would be an error [71]. Legislations are effective when used in conjunction with ethical principles, commitments in data use agreements (DUAs), and technical safeguards provided by the de-identification and anonymization process [52,72]. Stricter DUAs can be used to mitigate the loss of data quality that would otherwise be required if the technical process alone had to guarantee the privacy [48,73]. Current rules and regulations are seen by some authors as too soft to discourage attempts at re-identifying data, however, the same authors recommend consistency over severity in prosecuting the misuse of health data [71].

#### Accountability

The legal responsibilities and the ethical obligations are shared by all those involved in the collection or in the use of the data (ie, institutions or individuals) [10]. Research participants generally believe that anyone who uses their information, regardless of when and under which circumstances, share these responsibilities [50].

#### Institutional Review Boards

Review boards play an important role in the secondary use of health data. Although de-identified or anonymized data, in some cases, are not considered individually identifiable health information, research projects involving such data generally require IRB submission and approval. In these situations, the IRB assesses the information the subjects received, what they consented to, and whether the proposed research could be conflicting with their interests [31,72]. Eventually, if the IRB approves the project in question, it waives the obligation for informed consent [7,13,31].

### Experts’ Recommendations

The highest level of protection can only be provided by multidisciplinary approaches combining organizational, legal, ethical, and technical safeguards [10,59,72-74]. Relying exclusively on one of these aspects would be a mistake [75]. More information and training should be provided to researchers about privacy protection and about the risks associated with data sharing [60,69,74]. Numerous researchers express the necessity to review and update the current legal framework [10,31,56,59,71]. Many authors consider the ambiguity of the vocabulary and the misuse of terms as a problem that urgently requires a cooperative effort from the expert community [19,38,56,74]. When applying anonymization techniques, researchers generally recommend favoring privacy over data quality in the process of de-identification and anonymization [75,76].

## Discussion

### Principal Findings

The development and the application of privacy-enhancing techniques to health data has come to represent a research domain in its own right. This domain is growing rapidly, as demonstrated by the increasing number of publications and the arrival and geographical spread of new research groups. Researchers come from different disciplines and often have qualifications in several fields themselves. Computer science, biomedical informatics, and medicine are the most prevalent backgrounds overall; the main purpose driving the development and application of privacy-enhancing techniques to medical data is to facilitate biomedical research.

At the beginning of the 2000s, great hopes leaned on our abilities to develop technical safeguards that would unleash the potential of the secondary use of medical data. Almost 20 years later, our knowledge and competences have significantly improved, although every advance has come with new interrogations and challenges. Methods are still difficult to generalize or scale and inevitably alter the data quality, which can notably hinder its use for research. A successful exercise lessens the risk of re-identification while maintaining a sufficient level of data quality for research to be performed. In this aim, legal and contractual safeguards are essential and their use can be tailored (ie, made stricter or more lenient) to each situation to mitigate the technical limitations. The research community emphasizes that the different approaches (ie, organizational, legal, ethical, and technical) are complementary and necessary to provide an acceptable level of protection. What is an acceptable level of protection, however, is not easily defined. It varies both in the views of different experts and in the legislations of the different countries.

This work confirms and further illustrates the existence of a disconcerting confusion in the domain’s vocabulary affecting the understanding of the concepts at multiple levels. The vagueness and lack of consensus among the experts is worrying and requires actions. The life sciences research community is aware of this situation and is calling for clear and standardized definitions and for cross-border regulatory frameworks.

### Propositions

#### Clear Definitions

Appropriate use of the terms *de-identification* and *anonymization* should be promoted and incentivized. As a first step in this direction, the authors of this work suggest that future publications on the subject include definitions or state which definitions are referred to. Although not universal, clear definitions are provided in two major legislations on personal data protection (ie, the GDPR and the HIPAA) and should be used where appropriate.

The GDPR defines anonymous information as “an information which does not relate to an identified or identifiable natural person or to personal data rendered anonymous in such a manner that the data subject is not or no longer identifiable” [77]. It is an irreversible state. Accordingly, the term *anonymous* should not be used to describe the process of rendering data less identifiable, which is the prevailing representation of de-identification and anonymization in the biomedical literature. To refer to the concept of rendering data less identifiable, or to the techniques that are used in this aim, the GDPR defines the term *pseudonymization*: “the processing of personal data in such a manner that the personal data can no longer be attributed to a specific data subject without the use of additional information, provided that such additional information is kept separately and is subject to technical and organisational measures to ensure that the personal data are not attributed to an identified or identifiable natural person.”

Finally, the term *de-identification* comes from the American legislation where definitions are provided: HIPAA §164.514(a) and (b). Authors using this term in their publications should refer to these definitions.

#### Development of Clear Guidelines

In a manner that already exists in most clinical disciplines, international guidelines regarding privacy protection should be developed, agreed upon, and made widely available to the stakeholders in the field of biomedical research. These guidelines should clarify the concepts, the definitions, and the techniques, as well as their results and risks.

#### Improved Dissemination and Education

A striking result of this work is the lack of information dissemination and education at all levels. It is critical that the research community gains access to the appropriate information, definitions, and guidelines on the subjects of data privacy and data protection. The public and the media should benefit from this improved access and understanding. Building trust is essential for life sciences research to leverage today’s technological capabilities in accessing, sharing, and analyzing data. With this aim, information dissemination is key (see Textbox 4).

#### Limitations of This Work

There are several limitations to this work. As for any scoping review based on free-text searches, contributions may have been missed despite having maximized the search sensitivity. Privacy protection of health data is a rapidly evolving domain. Between the end of the scoping review and January 2019, 14 additional publications would have to be assessed for eligibility (ie, 114 vs 100).

The fact that the literature search was limited to MEDLINE introduces a strong but deliberate selection bias toward the domain of life sciences. Within life sciences, *genomics*, *personalized health*, and *precision medicine* may be overrepresented due to their growing popularity in recent years and their characteristic need for large amounts of sensitive data.

During the data collection, it was not possible to find the background of 7 authors. This represents 4.3% of the total author count (N=163), which should not impact the validity of the results.

#### Conclusions

Health data is increasingly produced and used. This wealth of information should not be left dormant as it represents a real potential to fuel research and improve medicine. Multidisciplinary safeguards (ie, ethical, organizational, legal, and technical) are required to guarantee the privacy of health data subjected to secondary use. Creating an overall trusted environment to leverage scientific research in life sciences is essential. It requires building on safe and strong foundations, to have processes and structures in place to enforce these foundations, and to communicate widely with the public and the media.

**Recommendations for future work.**

- Future publications should include definitions or state which definitions are referred to.
- Existing definitions proposed by major legislations (ie, the Health Insurance Portability and Accountability Act [HIPAA] and the General Data Protection Regulation [GDPR]) should be used where applicable.
- Global and specific guidelines should be developed to define the field of application, the process, the expected results, and the risk of the different technical approaches to privacy protection.
- Information dissemination and education should be improved across the research community for all stakeholders.

## Appendix

Multimedia Appendix 1 List of the 60 articles reviewed in this work.

## Abbreviations

DUA: data use agreement
GDPR: General Data Protection Regulation
GO FAIR: Global Open Findable, Accessible, Interoperable, and Reusable
HIPAA: Health Insurance Portability and Accountability Act
IRB: institutional review board
MBA: Master of Business Administration
MD: Doctor of Medicine
MeSH: Medical Subject Headings
NIH: National Institutes of Health
NLP: natural language processing
ORCID: Open Researcher and Contributor ID
PHI: protected health information
PRISMA: Preferred Reporting Items for Systematic Reviews and Meta-Analyses
[ti]: Title
[tiab]: Title/Abstract
