# Supplementary material for: Use and Understanding of Anonymization and De-Identification in the Biomedical Literature: Scoping Review
Source: J Med Internet Res. 2019 May 31;21(5):e13484. doi: 10.2196/13484 (PMC6658290; doi:10.2196/13484)
Supplement: Multimedia Appendix 1 [file jmir_v21i5e13484_app1.pdf]

Use and Understanding of Anonymization and De-Identification in the Biomedical Literature: Scoping Review

R. Chevrier, V. Foufi, C. Gaudet-Blavignac, A. Robert, C. Lovis

List of the 60 articles included in the review

1. PMID = {28358693}
2. PMID = {24370391}
3. PMID = {28903886}
4. PMID = {24412834}
5. PMID = {28481298}
6. PMID = {24502938}
7. PMID = {28382417}
8. PMID = {24859155}
9. PMID = {28433677}
10. PMID = {23842533}
11. PMID = {28693480}
12. PMID = {22893444}
13. PMID = {28423805}
14. PMID = {23590738}
15. PMID = {28441940}
16. PMID = {25187183}
17. PMID = {28756441}
18. PMID = {23405076}
19. PMID = {29295189}
20. PMID = {22287248}
21. PMID = {29039370}
22. PMID = {22692265}
23. PMID = {28011594}
24. PMID = {22563145}
25. PMID = {28913771}
26. PMID = {22081224}
27. PMID = {27577394}
28. PMID = {23646086}
29. PMID = {27172145}
30. PMID = {22195223}
31. PMID = {27199298}
32. PMID = {21943371}
33. PMID = {27256129}
34. PMID = {22168526}
35. PMID = {27322502}
36. PMID = {21169618}
37. PMID = {27130179}
38. PMID = {20385806}
39. PMID = {26567325}
40. PMID = {20442151}
41. PMID = {27410040}
42. PMID = {21041752}
43. PMID = {27104620}
44. PMID = {21373375}
45. PMID = {27454754}
46. PMID = {20678228}
47. PMID = {25991179}
48. PMID = {20113465}
49. PMID = {25552878}
50. PMID = {21347085}
51. PMID = {26713253}
52. PMID = {19745408}
53. PMID = {26385376}
54. PMID = {19567795}
55. PMID = {26711410}
56. PMID = {22478909}
57. PMID = {25911674}
58. PMID = {18579843}
59. PMID = {24333850}
60. PMID = {18560111}
